# Contemporary Theory and Research

**Published:** 1893-03-25

**Authors:** 


### Contemporary Theory and Research.

### CHOLERA INOCULATION.

Dr. Haffkine, of the Pasteur Institute, contributes to the *Fortnightly Review* a detailed account of his method of cholera inoculation. He begins by pointing out the prodigious power of procreation of the bacillus, instancing the bacillus of typhoid which, under circumstances not even the most favourable, engenders more than ten billions of its kind in 24 hours. While certain microbes are harmless, others are pathogenic or sources of contagious disease. They are capable of being cultivated in artificially prepared milieux, and transplanted to animals by injection. Aniline colours are employed to detect microbes, being absorbed by them with extraordinary intensity while the milieu remains colourless. Microbes do not, as a rule, act directly on the animal organism by their presence as one might be tempted to believe, but rather by the chemical substances they create, which are dangerous poisons for the organism. Now this production of dangerous substances does not constitute a function inseparable from the existence of microbes. For by modifying some of the conditions of existence of the little being, such as the degree of environing temperature, and, above all, the aëration and action of free oxygen, it is possible to transform a microbe into another resembling it in all points except that it does not produce poisonous substances, or, at any rate, in only infinitesimal proportions. The microbe thus modified is called "attenuated." When this is introduced into the body of an animal it produces but slight indisposition, yet renders the animal proof against the original microbe. Exalted virus, having many times the strength of the original, is produced by reinoculating live animals from those who have succumbed to the virus, until the limit of strength is reached. In 1883 Dr. Koch discovered the cholera microbe or "bacillus virgula," resembling a minute stick, sometimes straight, but generally bent. Length, ·002 millimetre; breadth, one-fifth of length. But it was found that cholera was incommunicable to animals, either because the microbe cannot live in the animal, or because the animal is insensible to the poison of the parasite. Dr. Haffkine saw reason to believe that the first hypothesis was the true one, and directed all his endeavours to habituate the microbe to live in a hostile milieu. For this purpose he prepared a liquid 75 parts broth and 25 parts serum of rabbit's blood. Into this he introduced the cholera microbes, and after a due interval transplanted them to a mixture 60 parts broth and 40 parts serum He continued the transplanting process until a new generation of microbes was formed, which, when introduced into pure rabbit's blood, prospered and multiplied. Introduced into living animals they produced a disease bearing a striking analogy to cholera in human beings. Meantime Dr. Hueppe had been experimenting on guinea-pigs, and found them susceptible to the cholera microbe. But though the guinea-pigs died of the disease, the microbe found in their bodies died also almost instantly, so that no advance could be made by further experiments. Dr. Haffkine found that if the microbes from the dead guinea-pig were exposed to the air, and the virus passed successively through from 25 to 30 guinea-pigs of various sizes, an exalted virus was obtained producing very remarkable effects. Subcutaneous injections of this exalted virus inflict on the animal at the place of injection a frightful wound; but when the animal has recovered from the wound no subsequent injection into the intestines, of cholera virus has any effect, although invariably fatal to non-inoculated animals. It was clear that this system of inoculation was unsuitable to human beings on account of the wound inflicted. Dr. Haffkine then has adopted the method of double vaccination: (1) with attenuated virus, prepared by exposure to air heated to a point of 39C. of the virulent microbes—this in order to prepare the organism; (2) with exalted virus. He tried the inoculation first on himself. In all more than one hundred persons have since submitted to it. The symptoms experienced are dull pain at the place of inoculation, slight fever, and headache. Many of the persons inoculated have since been exposed to cholera with impunity, but the experiment has not been tried on a sufficiently large scale to justify any definite conclusion at present. Dr. Haffkine is preparing to make extensive observations in those parts of the East where cholera is pandemic, and his discovery that the virus preserved in phenic acid is equally efficacious with living virus will no doubt greatly facilitate the progress of the experiment.

### MERCURIAL FLANNEL.

M. Merget has invented a medicated flannel which, enclosed in ticking or some thick cloth, is placed under the patient's cheek while sleeping, and emits mercurial vapours which are said to be an efficient substitute for mercurial friction. Experimenting on himself and his pupils, M. Merget finds that a large quantity of mercury is assimilated by this method, and he maintains even that the healing action of the friction is due solely to the absorption of the vapours arising in the process, and that the absorption by the skin, so long as it remains unchafed, is absolutely null.

